# Toxin and Antitoxin of and the Protective Inoculation against Bacillus Welchii

**Published:** 1917-12

**Authors:** 


***Toxin and Antitoxin of and the Protective Inoculation against Bacillus Welchii.*** By Carroll G. BULL and Ida W. PRITCHETT, of the Laboratories of the Rockerfeller Institute for Medical Research. Abstracted from the *Journal of Experimental Medicine*, July, 1, 1917.

After experimentation with five cultures of the Bacillus welchii the authors conclude that the infectious processes are local in character — few or no bacilli being found in the general blood stream.

Glucose broth cultures (the fluid as well as the full culture) cause almost immediate death when injected intravenously; but this result is not due to agglutinated bacterial emboli or acid intoxication. The action is hemolytic. Death may result from a massive destruction of red corpuscles.

Berkefeld filters reduce the hemolytic and poisonous effects of the fluid. Cultures in plain broth with sterile pigeon or rabbit muscle added are extremely toxic and are hardly lessened by filtration. The filtrates do not lose toxicity by neutralization with sodium hydroxide, but may do so when heated.

Carefully graded doses of this toxic filtrate in pigeons and rabbits produce active immunity. Blood from the immunized rabbits can neutralize the toxic filtrate *in vivo* and *in vitro*. Hence the filtrate has been considered as toxin and the immune serum as antitoxin, which neutralizes the toxin in multiple proportions, indicating that the latter possesses exotoxin properties. It also neutralizes the hemolytic as well as the locally injurious toxic constituent.

The authors conclude :

" Antitoxic serum prepared from a given culture of Bacillus welchii is neutralizing to the toxins yielded by the other four cultures of that microorganism. The antitoxin is protective and curative against infection with the spore and the vegetative stages of Bacillus welcii in pigeons. The limits of the protective and curative action are now under investigation ".

To quote further :

" According to our view, infection by Bacillus welchii, like infection by Bacillus tetani, essentially resolves itself into an intoxication, in which an exotoxin yielded by the multiplying organisms constitutes the chief danger. The two conditions differ, however, with respect to the local effects produced on the tissues, since the tetanus toxin does not possess inflamatory and necrotizing properties. The Welch bacilli grow more abundantly and produce wide destruction of tissue, in which process they are soon assisted by the usual pyogenic microorganisms, which quickly gain a foothold in the disorganized structures ".

